# Tracking the Vector of *Onchocerca lupi* in a Rural Area of Greece

**DOI:** 10.3201/eid1807.AD1807

**Published:** 2012-07

**Authors:** Domenico Otranto, Filipe Dantas-Torres, Elias Papadopoulos, Dušan Petrić, Aleksandra Ignjatović Ćupina, Odile Bain

**Affiliations:** Università degli Studi di Bari, Valenzano, Italy (D. Otranto, F. Dantas-Torres);; Aggeu Magalhães Research Centre Oswaldo Cruz Foundation, Recife, Brazil (F. Dantas-Torres);; Faculty of Veterinary Medicine, Thessaloniki, Greece (E. Papadopoulos);; University of Novi Sad Faculty of Agriculture, Novi Sad, Serbia (D. Petrić, A. Ignjatović Ćupina);; and Muséum National d’Histoire Naturelle, UMR 7205 CNRS, Paris, France (O. Bain)

**Keywords:** Onchocerca lupi, vector, Greece, scientific expedition, parasitology, eye, vector-borne infections, parasites, nematodes

During a hot Mediterranean summer, an expedition brought parasitologists from Brazil, France, Greece, Italy, and Serbia to a wooded area near Xanthi, Thrace, northeastern Greece, near the Turkish border, on the track of the vector of the little-known nematode *Onchocerca lupi*. The scientific purposes of the expedition blended then with stories of humans, animals, and parasites in this rural area.

## The Beginnings: What’s that Worm in the Turkish Blue Eye?

In the early months of 2010, Nermin Sakru, a microbiologist from the Medical School of Trakya University, Erdine, Turkey, contacted one of the authors (D.O.) seeking advice on how to identify a nematode extracted from the eye of an 18-year-old girl who had never traveled out of her native Trakya. Nematodes that might have caused such an infestation were many (*1*), including *Thelazia callipaeda*. This helminth, which infests carnivores and humans, has been studied for more than a decade at the University of Bari in southern Italy (*2**–**5*); this research was what led the Turkish colleague to establish original contact. The patient complained of pain and redness in the left eye and reported being bitten by a fly on the left eyelid in the evening (around 5:00 PM), ≈30 days before onset of symptoms. Pain caused by a biting insect was suggestive of infestation other than by *T. callipaeda*, which is transmitted by *Phortica variegata* (Diptera, Drosophilidae), an insect that gently feeds on the ocular secretions of its hosts during the pleasantly warm Mediterranean summers (*6*).

Some days later, the nematode was morphologically and molecularly identified as a little spirurid, *Onchocerca lupi*, known to infest dog eyes, inducing an acute or chronic ocular disease characterized by conjunctivitis, photophobia, lacrimation, discharge, and exophthalmia. At that time, this helminth infestation had never been reported in dogs in Turkey, and information on the biologic features of the nematode was still meager, despite its wide distribution in Greece, Germany, Hungary, Portugal, and Switzerland and the increasing number of reported cases (*7*). *O. lupi* nematode infestation in humans (*8*) and its biologic and pathogenic affinity with *Onchocerca volvulus*, the agent of river blindness, heightened the interest of D.O. and F.D.T. in the life cycle of this nematode. The idea of investigating the biologic features of *O. lupi* soon began to move across the convoluted pathways of their brains like larvae of *Oestrus ovis* (the nasal bot fly) migrating toward the central nervous system, the main decisional center of all animals!

## Organizing the Scientific Expedition: Paris and Antwerp

In the early autumn of 2010, D.O. and F.D.T. had the opportunity to work at O.B.’s laboratories at the Natural History Museum in Paris to morphologically describe dermal microfilariae of a filarioid of the genus *Cercopithifilaria,* isolated some months earlier from a dog in Sicily (*9**,**10*). Those days were short in sunlight, as fall reached Paris much earlier than southern Italy, making the laboratory not the coziest place for late shifts. The helminthology laboratory of the museum is a historically rich place where such eminent scientists as Alain G. Chabaud had described numerous nematodes of medical and veterinary concern for more than 30 years (*11**,**12*). Working in that small laboratory with simple, dated, yet handy, equipment led to inspiring discussions about prospective studies. By dealing with the zoonotic infestation by *O. lupi* nematodes, they realized how important this parasite species could be, even as a model for better understanding of *O. volvulus* pathogenesis. A major gap in the knowledge of this parasite species was with regard to its vector.

D.O., F.D.T., and O.B. supposed that, as in several other *Onchocerca* species, the potential vector of *O. lupi* could be a black fly (Diptera, Simuliidae) (*13*) or even a biting midge (Diptera, Ceratopogonidae) (*14*), so they decided to carry out a field study in an area where this parasite species is endemic. The choice for the best places to look for animal cases was not easily made as this infestation had never been reported in Italy, Brazil, or France. However, O.B. recalled that canine onchocercosis caused by *O. lupi* infestation was reported in the Chalkidiki peninsula, province of Thessaloniki, Greece (*15*), where E.P. has been active for 2 decades in veterinary parasitology.

Months later, during the annual meeting of the European Network for Arthropod Vector Surveillance for Human Public Health (Antwerp, April 2011), 2 Serbian entomologists (D.P. and A.I.C.) with expertise on black fly taxonomy and biology were hearing about this new parasite and the enthusiastic plans of an Italian researcher (D.O.) keen on studying its vector. The hypothesis of this scientific expedition blended then with stories of researchers and parasitologists in the years of the Yugoslav Wars (1991–1995) (*16*). Scientists around the table agreed that, sometimes, research activities do soothe physical and mental pains, helping to get wars out of people’s minds. Once back in Novi Sad (Serbia), D.P. and A.I.C. decided they would take part in the expedition with O.B., F.D.T., D.O., and E.P.

## In Thessaloniki on the Way to Xanthi

In June 2011, participants of the expedition team from the National History Museum in Paris (O.B.), the School of Veterinary Medicine of Bari (D.O. and F.D.T.), the School of Veterinary Medicine of Thessaloniki (E.P. and Socrates Ptochos), and the Faculty of Agriculture, University of Novi Sad in Serbia (D.P. and A.I.C.) received the first message in preparation for the expedition:

“The main aim of our expedition is to study the occurrence of *O. lupi* infested dogs and to identify the vector of this nematode. It will be a fieldwork whose protocol might require some adjustments according to the preliminary results. Skin and blood samples will be collected from dogs living in areas around rivers to diagnose *O. lupi* infestation. In the meantime, adult black flies shall be collected by dry ice baited traps and drop nets directly on dogs. During the study, samples will be processed and examined at night in accordance with the field activities.”

The expedition was partially funded by a pharmaceutical company (see Acknowledgements) and with a budget saved from a former (and different) project of the University of Bari and made available for this research. Because of economic constraints and the numerous commitments dictating the life of any academic, the project duration was fixed at 9 days—a terribly short time for such an ambitious task.

The first step toward retrieval of the developmental infective larvae in a putative vector collected in the field was to identify an area of *O. lupi* infestation. The first meeting took place in a clean, pretty hotel in the vicinity of Thessaloniki airport. While some of us (D.O., F.D.T., O.B.) arrived by plane, others (D.P., A.I.C.) arrived from Serbia after a many hours of driving. Almost nobody knew each other, and the first dinner, in spite of the tiredness, was the real kick-off meeting for the discussion and planning of the field activities. At the end of the first day, all team members were focused on their own duties and commitments in the expedition.

Early in the morning of the second day, after we had a chat over a coffee with A. Komnenou for better defining the localities where *O. lupi* infestations had been reported, the expedition moved to a wooded area near Xanthi. In ancient times, Thrace was considered the fourth continent, after Europe, Asia, and Africa, because of its great difference from the rest of Greece. Geographically, it belongs to the Balkans, with 5 major river systems and few safe anchorages. In this area, until late in the 20th century, population centers were formed in the foothills of the valleys, far from the mosquito- and pirate-infested marshy coastal lowlands.

Our hotel was located just outside Xanthi, on the way to Mount Koula near the small river Kosinthos. Most roads were constantly crossed by turtles (*Testudo graeca*) that carried ticks (*Hyalomma aegyptium*). This place was chosen for the small stream in a large, stony valley and for the overall environmental characteristics of the surroundings―bushes and oaks representing the optimal biotope for black flies (putative vectors of *O. lupi*).

**Figure 1:**
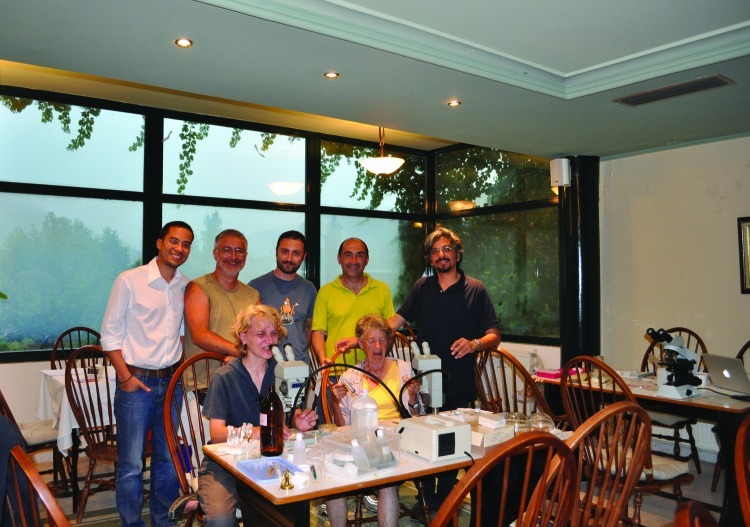
Expedition headquarters in large dining room of a hotel near Xanthi, Greece. Expedition team members (left–right): seated, Aleksandra Ignjatović Ćupina and Odile Bain; standing, Filipe Dantas-Torres, Dušan Petrić, Elias Papadopoulos, Socrates Ptochos, and Domenico Otranto.

We were the only guests at the hotel, and the owner allowed us to use a large dining room as our makeshift laboratory (Figure 1). We set up 3 optical microscopes and a stereomicroscope on 4 tables, together with slides, entomologic forceps, and ethanol necessary for checking, at night, samples collected during daytime.

## Kalimera, Xanthi: The Frontier Land

The expedition team members soon learned that Xanthi was the hometown of Democritus (c. 460 BCE–370 BCE), the ancient Greek philosopher who formulated the atomic theory for the cosmos. With a population of ≈55,500, this region is a frontier land. The main sampling area of the expedition was located around the boundaries of the small village (≈1,000 inhabitants) Amaxades (longitude 25°04′27′′E, latitude 41°07′12′′N, altitude 56 m), between Xanthi and Komotini. This is a traditionally agricultural area, with soil particularly suitable for tobacco cultivation. Most samples were taken at the cabin of veterinarian Triantaphyllos Papavasiliou, who had been a fellow student of E.P. at the University of Thessaloniki. As soon as the rumor of foreign academics visiting the village of Xanthi in search of some odd parasites in dogs’ eyes spread, a queue of old and young locals soon formed outside the cabin, with many more joining in the evening of the first day of field work. These were working people, tough in their faces as well in their hands, with deep, authentic eyes, not so different from those of southern Italians, or Serbians, or any others living where hard work shapes life paths. They were people suspended between their history and their present, and hardly the future, such as for their language, which is a mixture of Greek and Turkish. Nearly the entire Muslim population of Greece is concentrated in these villages, earning its living from a combination of livestock breeding (especially small ruminants) and agriculture. Animals are reared according to a semi-extensive system―grazing during the day and being corralled within stalls at night―with substandard hygiene and husbandry in most of the rural properties we had the opportunity to visit. Sheep and goats are kept for dairy purposes and are milked twice a day. Wolves represent such a threat in the area that farmers usually keep a number of shepherd dogs on their properties. Like their owners, dogs are usually malnourished and have a variety of diseases, most commonly parasitic (sarcoptic mange; tick, flea, and filarial worm infestations; gastrointestinal helminthiasis). We knew that dogs commonly feed on sheep and goat carcasses, a practice resulting in the transmission of pathogens that cause diseases such as coenurosis, hydatidosis, and toxoplasmosis. Human hydatidosis is one of the most prevalent zoonoses in this part of Greece (*17**,**18*).

While we collected samples from dogs, we also collected hematophagous insects by carbon dioxide (dry ice, NS-2 type) installed near the river stream close to our hotel, and by sweep nets, drop nets, and aspirators. Eventually, black flies, and other hematophagous insects of the *Culicidae*, *Psychodidae*, *Ceratopogonidae*, and *Tabanidae* families, were collected. Skin and blood samples were also collected from 21 animals of different ages and sexes. Their owners collaborated with the sample collection, providing information on where their animals were kept and their habits.

Six dog samples were positive for *Dirofilaria immitis* microfilariae, which fit with the occurrence of potential vectors (*Ochlerotatus caspius*, *Culex pipiens*, *Anopheles maculipenis* s.l.) of this filarioid in this area. On the fourth day of sampling, with only 2 days of work left, no dog samples were positive for *O. lupi*. Nonetheless, black flies were captured early in the morning and in the evening by netting directly on some horses grazing around the hotel and by dry ice–baited traps on the riverbanks. At noon, when everything seemed to be lost, E.P. received a phone call from Dr. Papavasiliou, who was about to depart to an Aegean island for holidays. A farmer had just called him complaining of blindness in his dog, and Dr. Papavasiliou promptly suggested that he visit the expedition’s headquarters for a clinical check and sample collection; he managed this just a few seconds before being advised by the flight crew that “all mobile phones must be switched off during takeoff.”

## In *Onchocerca lupi*’s Lair

**Figure 2:**
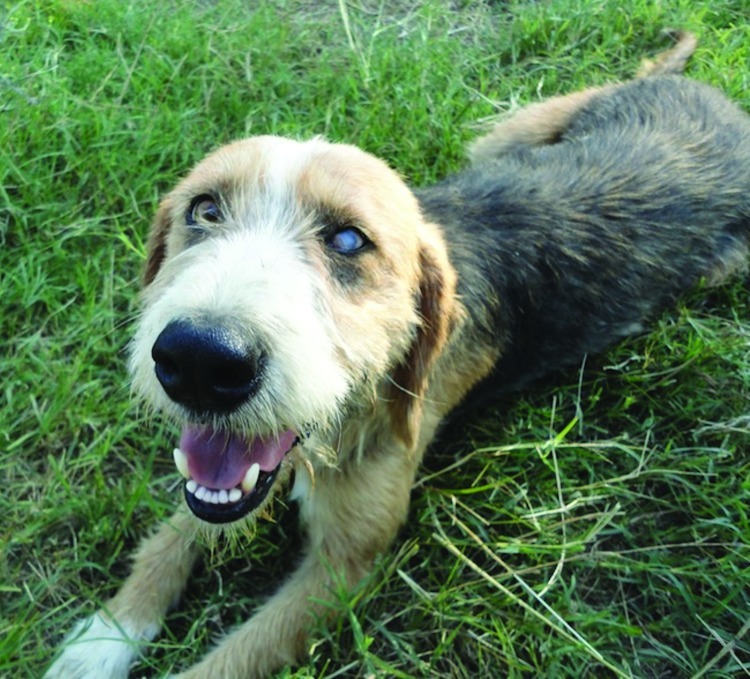
Dog with keratitis and uveitis that was found to be positive for *Onchocerca lupi* nematodes by parasitologic examination.

The dog’s owner, a shepherd, arrived at the hotel accompanied by his son and carrying a small, febrile animal. After the clinical check, skin and blood samples were collected and processed within an hour at the makeshift laboratory. The skin sample was positive for *O. lupi* first-stage larvae, prompting the researchers to immediately visit the farm where the animal lived. It was a roasting hot afternoon (June 24, 2011) when the expedition team reached the rural area close to the village of Komotini to take samples from other dogs living in proximity to the animal with the positive sample. The shepherd’s family seemed pleased to host us and very eager to assist with research activities. The sheep flock was watched over by both father and son. In the same farm, another animal, with visibly impaired eyesight, keratitis, and uveitis, was found positive for *O. lupi* (Figure 2).This seemed the right time (the only time left) for exposing the collected black flies to feed on the 2 infested dogs in World Health Organization cages (used for insecticide resistance testing). Small glass tubes of entomologic aspirators were used.

Therefore, 4 glass tubes with ≈20–30 black flies each were held on the shaven skin of the head of the positive dogs for 30 minutes to allow them to feed. Out of 4 black flies species, 3 (*Simulium velutinum*, *S. reptans*, and *S. pseudequinum*) fed on the dogs. Afterwards, the blood-fed black flies were kept in the same glass tubes with proper cotton tap impregnated with sugar water solution, ready to be brought to France for dissection and examination for developing larvae of *O. lupi* at +7, +14, and +21 days. Only time would tell the success of the expedition. In heavy rain, the team drove back to Thessaloniki to rest before taking the plane home.

## Epilogue

Some weeks after the end of our field activities, O.B. wrote to the expedition team that no developing larvae were detected in the dissected black flies. This should have been expected because of the low number of fed black flies dissected (n = 11). From the beginning of their adventure, researchers knew the inherent difficulties, essentially due to the short time available to look for *O. lupi*–infested dogs, to collect black flies, and to try and feed them on infested animals. It was a challenging task to be accomplished in 9 days. However, their motivation remained high throughout the entire expedition. It was something between irrationality and strong will to learn the life cycle of this scarcely known parasite. The uniqueness of the experience was linked to the fact that, because of the crisis affecting the economies mostly in Greece and Italy, and over the past 20 years, in Serbia, funds for basic research have diminished and are only used for applied research on parasites with major impact on human and animal health. Although acknowledged as a parasite of zoonotic importance in Turkey (*8*) and Tunisia (*19*), *O. lupi* so far has minimal relevance for human health. Nonetheless, the closely related *O. volvulus*, causative agent of human river blindness, is an important cause of visual impairment, affecting >17.7 million globally and remains of major public health importance, especially in developing countries (*20*).

During those days, we learned that the originality of basic research should reside in walking any possible pathway, even the most narrow, tortuous tracks that could lead to medical science discoveries. This principle, beyond any other rational reason, should be the trigger of scientific research.
